# Improving and standardizing concussion education and care: a Canadian experience

**DOI:** 10.2217/cnc-2018-0007

**Published:** 2018-10-24

**Authors:** Faraz Damji, Shelina Babul

**Affiliations:** 1 Graduate Program in Rehabilitation Sciences, Faculty of Medicine, University of British Columbia, Vancouver, BC, V6T 1Z3, Canada; 2 Clinical Associate Professor, Department of Pediatrics, University of British Columbia, Vancouver, BC, V6H 3V4, Canada; 3 Associate Director, BC Injury Research & Prevention Unit, BC Children's Hospital, Vancouver, BC, V6H 3V4, Canada

**Keywords:** Canada, concussion, education, knowledge translation, mild traumatic brain injury, public health, standardization

## Burden of concussion in Canada

Concussion, also known as mild traumatic brain injury, is an acute urgent public health issue that has the potential for serious long-term consequences if not recognized immediately. It is a complex injury affecting the brain both structurally and functionally [1–3] as a result of neurological deficits that occur when the brain is jostled inside the skull [4]. This particular injury is unique in that no two concussions are alike, as each patient responds differently. One may take a significant hit and recover uneventfully in days, while another may take a seemingly minor hit and yet still be recovering months later. There are no diagnostic tools currently available to assess a concussion and, as a result, diagnosis is not an exact science. Every concussion needs to be treated on a case-by-case basis, usually taking into consideration previous history, circumstance of injury and presenting signs and symptoms.

Quantifying the burden of concussion in Canada is a difficult task due to the lack of consistent reporting that is collected across the country. The Canadian Institute for Health Information reported that there were 645 injury hospitalizations due to sport-related concussion across Canada in 2016–2017 [5]. From a provincial perspective, of the total cases of sport-related head injuries that presented to emergency departments in Ontario and Alberta in the 2016–2017 fiscal year, 94% of the admitted cases (∼481) were diagnosed as concussions [5]. In the province of British Columbia, as captured by the Discharge Abstract Database for hospitalizations, there were 2785 concussion hospitalizations from all causes over a 5-year period from 2012/2013 to 2016/2017, averaging 557 hospitalizations per year [6]. While sport-related concussions are most prominent in the media, other common causes of concussions include falls and motor vehicle crashes.

From an economic perspective, the burden of concussion is also staggering. In British Columbia in 2013, US $3.3 million was spent on direct healthcare costs for concussion-related hospitalizations, for all ages [7].

The concussion data that are available does not truly convey the magnitude of the problem at hand. The numbers do indicate that the highest incidence of concussion is observed among youth below 18 years of age and that prevalence is growing at a rapid rate [8,9]. It is also worthwhile to note that these numbers are an underestimation as, in many cases, they do not include those individuals seen by their family physicians or through walk-in clinics, or take into account concussions that went unrecognized and unreported.

### Current state of concussion knowledge & practice

The first International Symposium on Concussion in Sport was held in Vienna in 2001. The aim of this conference was to ‘provide recommendations for the improvement of safety and health of athletes who suffer concussive injuries in ice hockey, football (soccer) and other sports [10]. A range of experts in the field were invited to inform others on their knowledge and understanding surrounding concussion-related issues. A document summarizing the main findings and the agreement position reached was published by a small group of experts that has now become known as the Concussion in Sport Group (CISG). This statement was groundbreaking in that it presented a single definition for ‘concussion in sport’ and offered a stepwise return-to-play protocol. The intended audience was anyone involved in the care of injured athletes at all levels from recreational to professional.

Since Vienna, several other international meetings have transpired along with the flux of new concussion research. Scientifically sound statements have been released following each of the conferences that are built on previously established principles and have furthered the conceptual understanding of sport-related concussion. The 5th Consensus Statement on Concussion in Sport, released in 2017 by the CISG, is currently recognized as the gold standard in concussion care [11]. It presents evidence-based recommendations that are meant to assist in maintaining a high standard of concussion care globally.

Concussions can affect anyone regardless of their age or which activities they are engaged in, yet many people do not understand what a concussion is or the potential it has to disrupt an individual's life. There is a disconnect between knowledge and practice as concussions are much more serious than the old adage of ‘having your bell rung’. From medical practitioners to coaches, parents and school professionals, everyone has the responsibility to immediately recognize, accurately diagnose and treat, and appropriately manage this precarious injury.

A 2014 study by Stoller *et al*. revealed that only some and not all physicians in Ontario are aware of the existence of a consensus statement on concussion in sport [12]. Carson *et al*. in 2016, found that Canadian sport and exercise medicine physicians are aware of the tools developed by the CISG and view them with high regard, yet this knowledge did not translate into consistent practice in the clinical setting [13]. Both the aforementioned studies speak to the need for strengthening knowledge translation activities that target medical practitioners. Patients are at risk of receiving inconsistent guidance about concussion from their healthcare providers, leading to confusion and the potential for longer recovery periods and poorer health outcomes.

The need for improved concussion education stems well beyond the arena of healthcare. It is imperative that other individuals who may potentially interface with concussion, such as athletes, students, parents, coaches and school personnel are also provided with much-needed concussion education. A federal survey commissioned by the Public Health Agency of Canada in 2017 found that approximately half of Canadians reported having little to no knowledge about concussions, and do not know where to find information on how to prevent one [14].

Among surveys of various athlete cohorts by researchers, continuing to play while symptomatic following a concussion is a common finding [15,16]. This implies that athletes are not properly educated about safe and appropriate behavior to minimize long-term brain damage. A study involving professional football players from the Canadian Football League found that 82.1% of those who believed they had suffered a concussion did not seek medical attention at the time of injury [17].

Coaches, who play a pivotal role in sports organizations and who have a direct influence on athletes, although generally well informed about concussion, also display some gaps in knowledge [18]. Among Canadian coaches of female youth hockey for example, information specific to the mechanism of injury, sign and symptom identification, and emotional symptoms would be particularly useful to address these poorly understood areas [19]. Evidence shows that key messages from published concussion guidelines such as appropriate return-to-play decisions have not reached many coaches, who instead hold misconceptions [20].

The education sector needs to ensure standardized and developmentally appropriate return-to-school practices in primary and secondary schools, which currently is not in place [21]. Knowledge deficits have been observed among educators in areas such as the description and identification of concussions, as well as the implementation of interventions in the classroom [22].

Despite the deficiencies in the current state of concussion knowledge and practice in Canada, it is important to note that that we have come a long way in the last decade, and that work toward national standardization of concussion response and management is underway.

### Recognizing the importance of concussion & moving toward a harmonized approach

In February 2015, the conference of Federal/Provincial/Territorial Ministers responsible for Sport, Physical Activity and Recreation was held in Prince George, British Columbia [23]. Officials in attendance were directed to establish a Federal/Provincial/Territorial Working Group on Concussions in Sport, that served to work toward developing a harmonized approach to address the issue of concussions. The Working Group was comprised of Federal/Provincial/Territorial governments, the national sport sector, the health sector and the education sector.

Later that year in November, Prime Minister Trudeau released a mandate letter for the Minister of Sport and Persons with Disabilities. In terms of concussion, priority was placed on working with the Minister of Health and the Public Health Agency of Canada to support a national strategy to raise awareness for parents, coaches, and athletes on concussion treatment.

In June 2016, the  Federal/Provincial/Territorial Ministers responsible for Sport, Physical Activity and Recreation reconvened in Lethbridge, Alberta [24]. At this conference, the liberal government formally recognized concussion as a significant public health issue. This recognition served as an important stimulus that set the stage for future governmental efforts directed toward enhancing the quality of life for those who have sustained a concussion.

In the months following the conference in Lethbridge, Canada's Minister of Sport pointed out how there is a lack of a common approach to address concussions. In terms of concussion protocols in Canada, great variance is evident between sports across the country. The Minister emphasized the need for a unified policy that allows concussions to be addressed in a comprehensive and consistent manner, regardless of when and where they occur. It is the hope that having a protocol in place will increase the likelihood of detecting concussions. This is imperative because, when concussions are not recognized or go undetected, the brain is not given the appropriate time to heal and recover, therefore possibly inducing further damage. Having a common approach would synthesize all of the work being done across the country in various areas, bringing it together to build a comprehensive strategy that amalgamates the best available evidence in the field.

In October 2016, the federal government provided $1.4 million for the Public Health Agency of Canada to accelerate the realization of a Pan-Canadian Concussion Strategy as mandated by the Prime Minister. Parachute Canada, a national nonprofit dedicated to reducing preventable injuries, with support from the Public Health Agency of Canada, led the development of harmonized concussion management guidelines and protocols. Parachute also established a Concussion Expert Advisory Committee which comprised of leading experts, including physicians and researchers in the field of concussion and traumatic brain injuries.

While this work was underway, His Excellency the Right Honourable David Johnston, Governor General of Canada, hosted a timely conference on concussions in sport at Rideau Hall in December 2016. The conference acted to raise awareness of the issue and to ensure all Canadians, especially the young, can safely and confidently participate in sports. The conference brought stakeholders from all sectors together fostering advanced collaboration while in the beginning stages of developing a national approach.

The Canadian Guideline on Concussion in Sport, developed by Parachute's Concussion Expert Advisory Committee, was released on July 28, 2017 [25]. Based on the evidence presented in the 5th International Consensus Statement on Concussion in Sport, these guidelines include best practices for the evaluation and management of athletes with a suspected concussion. The key areas addressed in this document include preseason education, head injury recognition, onsite medical assessment, medical assessment, concussion management, multidisciplinary concussion care and return to sport. It is intended for use by athletes, parents, coaches, officials, teachers, trainers and licensed healthcare professionals. As of June of 2018, 42 of the 56 national sport organizations in Canada have committed to implementing these protocols [26].

In addition to the development of the Canadian Guideline on Concussion in Sport, Parachute was also tasked with the development of an accredited online continuing education course for medical professionals. Parachute partnered with the BC Injury Research & Prevention Unit, BC Children's Hospital, in the development of the Concussion Awareness Training Tool (CATT) for Medical Professionals, which is an e-learning course that is free-of-charge, available in both English and French, and eligible for credits with the Maintenance of Certification (MOC) program through the Royal College of Physicians and Surgeons of Canada. Originally launched in 2013, the new course, which relaunched this past June, 2018, had significant updates. The revised version shifted its original focus from recognition and diagnosis to concussion treatment and management. Areas addressed in this revised version include how to effectively assess a patient's concussion situation within the initial hours post injury, how to optimally manage concussion care during the first 2–4 weeks post-injury, and how to identify when referral to specialty care is required. The content for the CATT Medical Professionals course is evidence-based and aligns with the 2017 recommendations from the consensus statement as well as other new and emerging resources. The Concussion Awareness Training Tool (www.cattonline.com) platform also provides education and resources to other audiences including parents, players, coaches and school professionals.

The passing of Rowan's Law in Ontario in March of 2018 was also a significant step forward in harmonizing concussion care. This was the first piece of legislation in Canada addressing concussion. This law arose after the death of Rowan Stringer, a 17-year-old rugby player who experienced repeated concussions culminating in Second Impact Syndrome. The law aims to improve concussion protocols in Ontario youth sports, by making concussion education, prevention and management practices compulsory, with the hope that such a tragedy does not happen again in the future.

Clearly, Canada has taken a proactive stance on the burden of this injury. In a rather short period of time, the landscape on concussion care in Canada has shifted to a more homogenous and evidence-based approach. Health Canada has called it *“an essential first step in Canada's approach to managing concussions”*, but also acknowledges that there is more work to be done [27]. All of the efforts mentioned thus far are contributing factors to the ongoing and coordinated efforts of federal, provincial and territorial governments to implement harmonized concussion protocols and practices. Though progress being made is promising, more work is required to address the need for improving and standardizing concussion education and care.

### Other catalysts for concussion standardization

Alongside the efforts put forth by the Government of Canada to address the issue of concussion, other initiatives have taken place in order to achieve the same goal of achieving standardization and ultimately keeping Canadians safe and healthy.

As mentioned, CATT is a series of online educational modules and resources that seeks to standardize concussion recognition, diagnosis, treatment and management, and is based upon the established principles of the Consensus Statement on Concussion in Sport. CATT strives to clarify the misconceptions among the general public regarding concussions by consolidating the latest, evidence-based information and presenting it in a way that is user-friendly for all audiences.

The CATT offers a completely individualized approach to understanding concussions based on what lens you are approaching from; medical professional, coach, parent or caregiver, player or participant, or a school professional. Based on an individual's selection, they may access an e-learning course and additional resources such as journal articles, informative handouts, protocols, guidelines and videos that are relevant to their needs. A new module for workers and workplaces is currently being developed and is expected to be released in the near future. The tool is freely available and can be accessed at any time. As the research and evidence on concussions is constantly evolving, updates are continuously being made to reflect the changing landscape of concussion. All e-learning courses have been evaluated and shown to be statistically significant in terms of changing knowledge and practice.

In June 2016, the CATT online course for coaches was mandated by BC Hockey to be completed by all team officials (∼14,000) prior to the start of the 2016–2017 hockey season. Evaluation of this policy change demonstrated that BC Hockey team officials reported a significant increase in concussion knowledge following CATT training and that they were better prepared to deal with an on-ice incident during the hockey season.

CATT has been disseminated provincially, nationally and internationally. To date, approximately 40,000 individuals have completed the CATT training with over 17 schools and sporting associations mandating concussion training in British Columbia.

Another major initiative worth noting is the body checking policy change introduced by Hockey Canada in 2013. This policy delayed the introduction of body checking until the Bantam age category (ages 13–14 years) in youth ice hockey, to reduce injury risk. This rule change has had a meaningful public health impact resulting in a 50% reduction in injury risk and a 66% reduction in concussion risk among Pee Wee (ages 11–12) players in Alberta, Canada [28]. Following this change at the national level, several provincial organizations in Canada disallowed body checking in the nonelite Bantam level (lower 60–70% division of play). Evidence suggests a 58% lower risk of game-related concussion in these nonelite Bantam leagues where local policy disallowed body checking [29].

Researchers focusing on mild traumatic brain injury have also directed their efforts toward examining blood biomarkers and neuroimaging techniques. This is an emerging area that has the potential to further our knowledge and ability to care for patients diagnosed with concussion. Current assessment of concussion includes symptom reporting, cognitive, neuropsychological and functional testing. Though useful immediately and in the short-term following injury, these measures are not particularly good at detecting differences among those individuals in the long term [30,31]. Identifying blood biomarkers and neuroimaging techniques would give an index of the underlying neuropathology, allowing quantification of the impact of an injury and trajectory of recovery. This information could advance our understanding of differences among patients, moving us toward a more individualized approach to treatment. A number of publications have identified biomarkers that reveal alterations in white matter, connectivity and neurophysiology [32–34]. Data from imaging studies of brain structure and function indicate that techniques such as diffusion tensor imaging and functional magnetic resonance imaging have a role in discovering concussion-related neuropathology [35]. Further research is currently being conducted in these areas to further our understanding and knowledge.

Recently, a group of scientists from the University of Calgary in Alberta have published their findings on a new device that is able to accurately determine if someone has suffered a concussion with a simple blood test [36]. The device is a handheld immunosensor with built-in electrodes capable of detecting key biomarkers. This research is still in its infancy as a diagnostic tool, but lends to the body of research that is currently underway examining such a complex yet treatable injury. Although much more research is needed, it has the potential to improve a medical professional's ability to diagnose by reducing the need to solely rely on judgement, offering an objective measure that is readily available.

Work is also happening in many other areas and researchers are constantly pursuing new ways to understand the burden of concussion and the impact it has on an individual's quality of life. Engineering efforts such as accelerometers placed in mouth guards and helmet technology are just some of the innovative technological advances being made in an effort to reduce the short- and long-term effects of concussions.

## Conclusion

Given the incidence rates of concussion, this acute public health issue has left many searching for answers to what can be done to avoid the dangerous risk of post-concussion syndrome. In turn, this has led to a surge in new and sophisticated research investigating the complexity of the brain and examining ways to protect it. The research findings will be the driving force behind the continued evolution of concussion management strategies. These strategies have gone from a one-size-fits-all approach, supported by primarily anecdotal evidence, to an increasingly individualized and evidence-based approach. Although our current practices are far superior to what was done in the past, there is still plenty of room for further enhancement in the coming years. With regard to sports, we have observed the beginnings of a culture shift toward prioritizing brain health over athletic competition and pushing to no avail. This is likely to become even stronger with more evidence on the perils of concussive injuries.

Concussion injuries have had a great impact on the Canadian population. This invisible epidemic is devastating to people as it may severely diminish an individual's quality of life if not recognized immediately and managed appropriately. Unfortunately, the prevailing knowledge gap across audiences who may be associated with concussion has made it a challenge to access information that is accurate, reliable and trustworthy. Yet, Canada has made remarkable progress in improving the standard of concussion education and care at the federal, provincial and territorial levels. This work has brought us one step closer to having a harmonized approach to concussion education, treatment and management. The key to addressing the need for standardized concussion care in Canada is through concerted knowledge translation efforts [37]. We have definitely made progress in recent years, but still have a long way to go.

## Future perspective

The field of traumatic brain injury and concussion is evolving, and our knowledge and understanding of the complexities of the brain is growing. Over the course of the next 5–10 years, we may be able to diagnose a concussion more accurately with clear diagnostic tools, and the guidance for patients and their treatment and management may be more individualized for complete recovery. We will strive to understand why two individuals respond and recover from a concussion so differently. In the meantime, we will continue to educate and raise awareness on the importance of this invisible epidemic while further examining the intricacies of the brain, investigating practical treatment modalities and researching effective preventative measures.
